# Mixed adenoneuroendocrine carcinoma of the distal bile duct: a case report

**DOI:** 10.1186/s40792-020-00921-x

**Published:** 2020-07-06

**Authors:** Takashi Maeda, Kyohei Yugawa, Nao Kinjo, Hiroto Kayashima, Daisuke Imai, Koto Kawata, Shinichiro Ikeda, Keitaro Edahiro, Kazuki Takeishi, Tomohiro Iguchi, Noboru Harada, Mizuki Ninomiya, Shohei Yamaguchi, Kozo Konishi, Shinichi Tsutsui, Hiroyuki Matsuda

**Affiliations:** 1 Department of Surgery, Hiroshima Red Cross Hospital and Atomic-bomb Survivors Hospital, 1-9-6 Senda-machi, Naka-ku, Hiroshima, 730-8619 Japan; 2 Department of Surgery and Science, Graduate School of Medical Sciences, Kyushu University, 3-1-1 Maidashi, Higashi-ku, Fukuoka, 812-8582 Japan

**Keywords:** Neuroendocrine neoplasm, Mixed adenoneuroendocrine carcinoma, Immunohistochemistry

## Abstract

**Background:**

Mixed adenoneuroendocrine carcinoma (MANEC) of the common bile duct (CBD) is very rare, with only 10 reported cases. Here, we report a case of MANEC of the distal bile duct (DBD) that was surgically resected under a diagnosis of cholangiocarcinoma (CCA).

**Case presentation:**

A 60-year-old male had epigastric pain and was admitted to our hospital for the treatment of a suspected CBD stone. Upon admission, laboratory findings revealed elevated hepatobiliary enzymes including serum aspartate aminotransferase, serum alanine aminotransferase, serum glutamyltransferase, and serum alkaline phosphatase. Both carcinoembryonic antigen and carbohydrate antigen 19-9 were negative. Computed tomography (CT) showed dilation of the CBD. Endoscopic retrograde cholangiopancreatography (ERCP) showed circumferential stenosis and a 5-mm elevated lesion in the DBD. Brush cytology showed atypical ductal cells, indicating adenocarcinoma (AC) of the DBD. Under a diagnosis of CCA of the DBD, a subtotal stomach-preserving pancreaticoduodenectomy was performed. Neither peritoneal dissemination nor lymph node metastasis was found. Microscopically, the lesion was seen to be composed of predominantly well-differentiated tubular AC in the superficial layer of the tumor, admixed with neuroendocrine carcinoma (NEC) in the deeper portion, indicating a diagnosis of MANEC of the DBD. After immunohistochemical staining, NEC components were positive for synaptophysin and CD56 and were for SSTR2, SSTR5, and mammalian target of rapamycin (mTOR). Three months postsurgery, postoperative adjuvant chemotherapy with S-1 was started. More than 3 years postsurgery, he is alive without recurrence.

**Conclusions:**

MANEC is highly malignant, progresses rapidly, and has a poor prognosis. Preoperative diagnosis is difficult; therefore, identifying NEC components by immunohistochemical staining using resected specimens is important.

## Background

Mixed adenoneuroendocrine carcinoma (MANEC) of the common bile duct (CBD) is extremely rare, with only 10 reported cases thus far [1–10]. The characteristics of MANEC remain poorly understood, and making an accurate preoperative diagnosis of biliary MANEC is extremely difficult. The prognosis of biliary MANEC is poor; however, treatment strategies of MANEC are not well established. The present study reports the case of a 60-year-old male with MANEC of the distal bile duct (DBD) that was initially diagnosed and surgically resected as cholangiocarcinoma (ACC).

## Case presentation

A 60-year-old male had epigastric pain and was admitted to our hospital for treatment of a suspected CBD stone. Past medical history was only hyperlipidemia. Mild tenderness in the upper abdomen was noted during physical examination. Laboratory findings on admission were as follows: white blood cell count, 6600/μl; hemoglobin, 14.0 g/dl; platelet count, 28.9 × 10^4^/μl; C-reactive protein, 0.35 mg/dl; total bilirubin, 2.0 mg/dl; direct bilirubin, 0.4 mg/dl; serum aspartate aminotransferase, 307 IU/l; serum alanine aminotransferase, 409 IU/l; serum glutamyltransferase, 932 IU/l; and serum alkaline phosphatase, 534 IU/l. Regarding tumor markers, carcinoembryonic antigen was 1.7 ng/ml (normal range < 5.0 ng/ml) and carbohydrate antigen 19-9 was 9.6 U/ml (normal range < 37.0 ng/ml). Viral markers for hepatitis, including hepatitis B surface antigen and hepatitis C viral antibody, were negative.

**Fig. 1:**
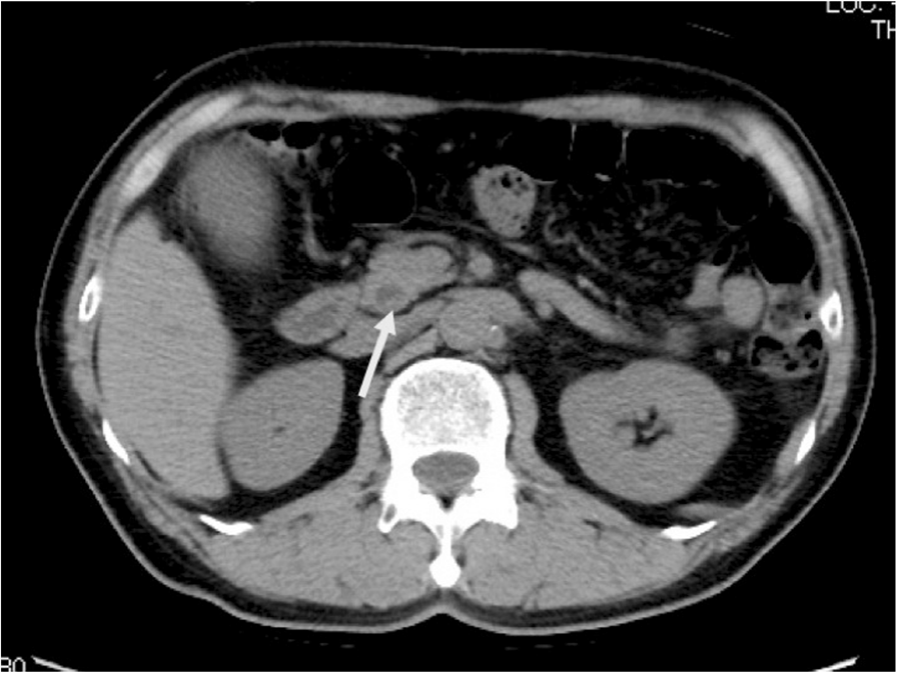
Abdominal contrast-enhanced CT showed the slightly dilated CBD and a high-density spot (arrow) in the DBD, suggesting CBD stone

**Fig. 2:**
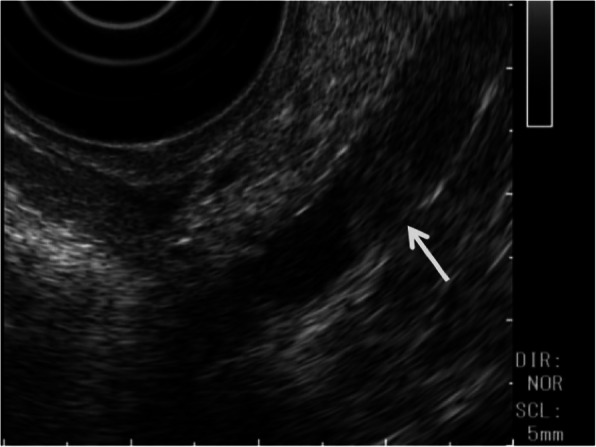
Endoscopic ultrasonography demonstrated an elevated lesion (arrow) of DBD

**Fig. 3:**
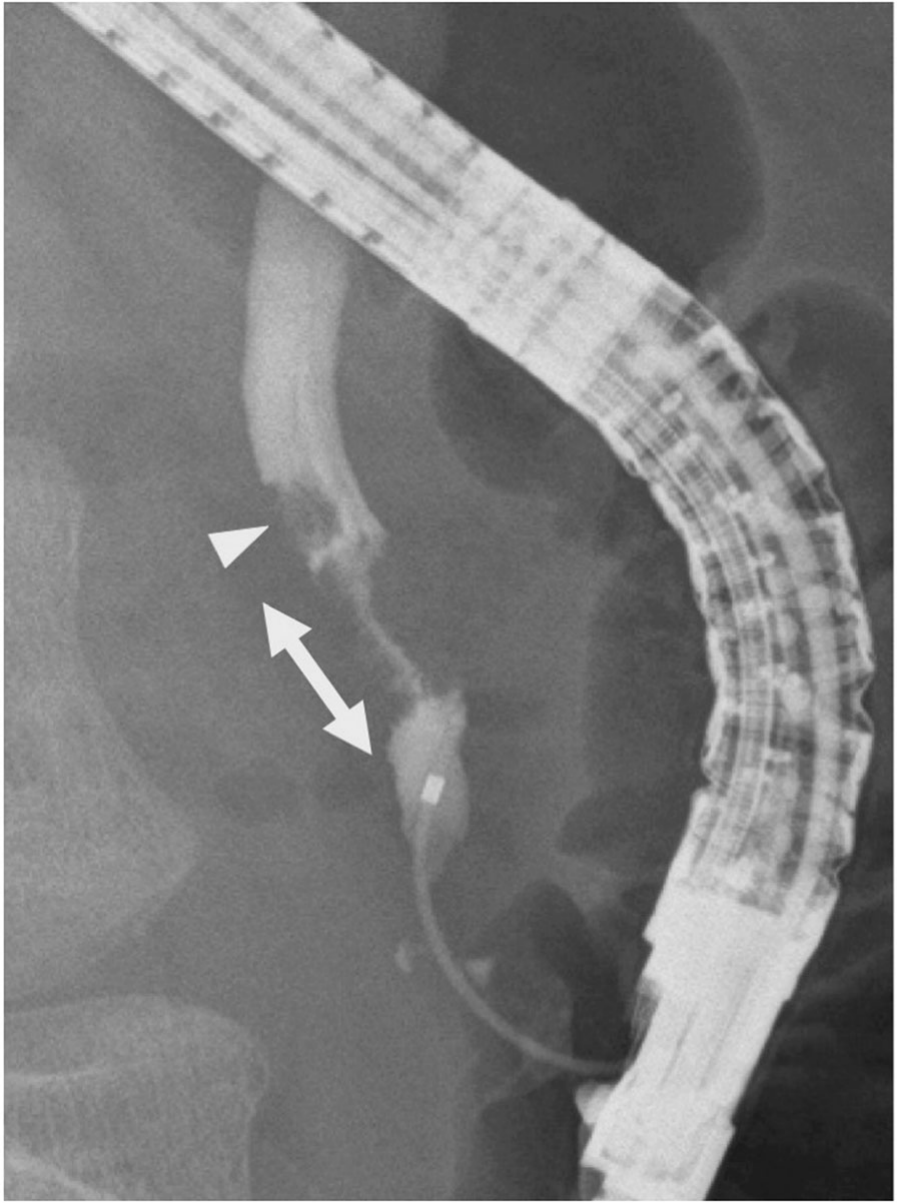
ERCP revealed a circumferential stenosis 11.8 mm distal from the ampulla of Vater (double arrow), and a 5.1 × 6.5-mm irregular-shaped elevated lesion (arrowhead) of the DBD

Abdominal contrast-enhanced computed tomography (CT) showed a slightly dilated CBD and a high-density spot in the DBD, suggesting a CBD stone (Fig. 1). Endoscopic ultrasonography demonstrated an elevated lesion on the DBD (Fig. 2). Permeation to the pancreatic parenchyma or to the outside of the bile duct wall was unclear. Endoscopic retrograde cholangiopancreatography (ERCP) revealed a circumferential stenosis 11.8 mm distal from the ampulla of Vater and a 5.1 × 6.5 mm irregularly shaped, elevated lesion on the DBD (Fig. 3). A double pig-tail catheter (7 Fr, 6 cm) was inserted in the bile duct. Brush cytology showed atypical ductal cells, indicating adenocarcinoma (AC) of the DBD.

**Fig. 4:**
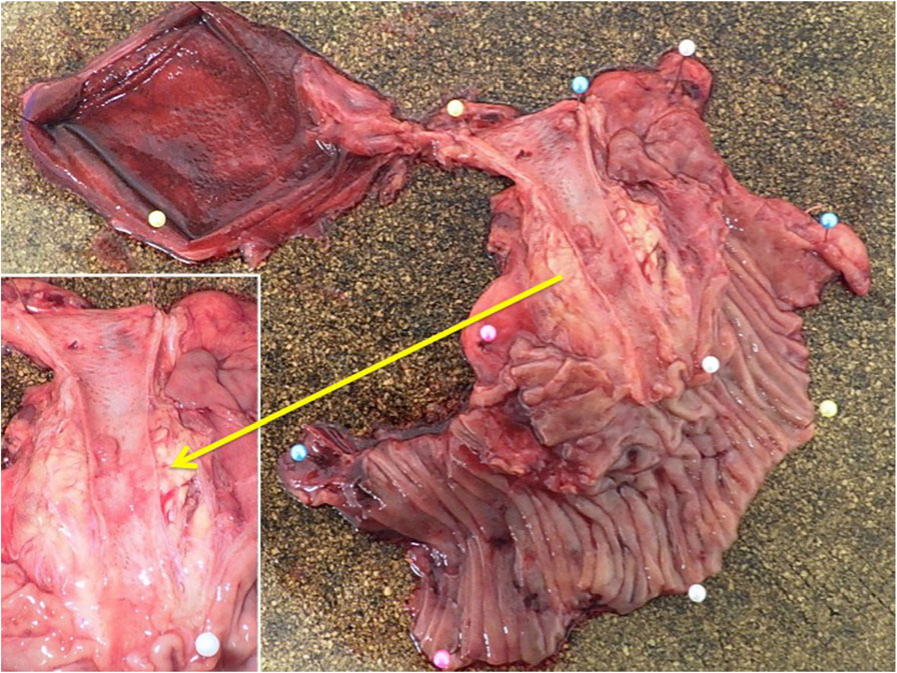
Macroscopic examinations of tumors usually reveal a nodular, infiltrating, or polypoid mass (arrow)

**Fig. 5:**
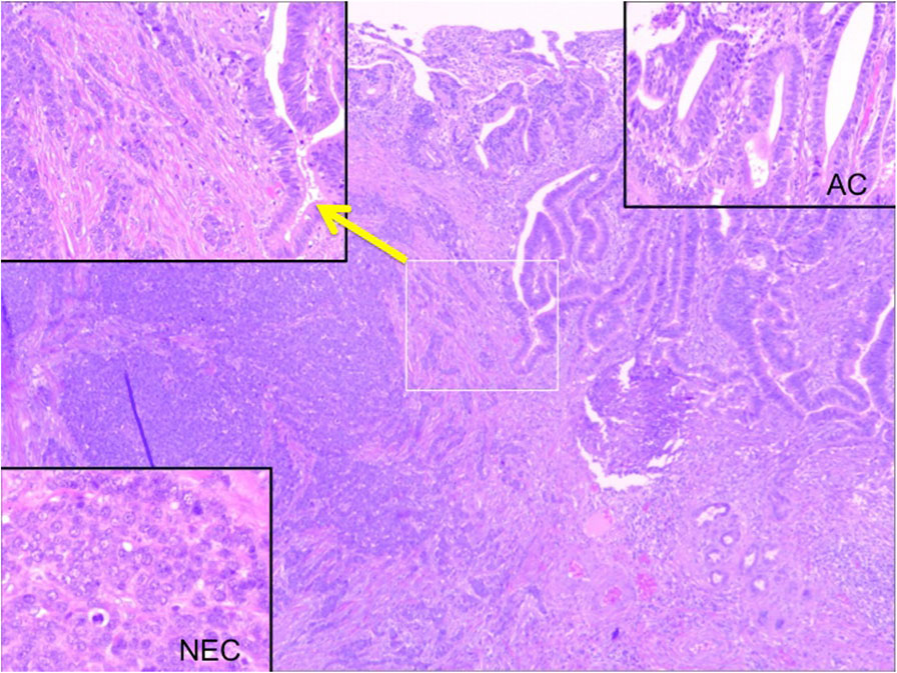
Microscopically, the tumor was composed of predominantly well-differentiated tubular AC in the superficial layer, admixed with NEC in the deeper portion, indicating MANEC of the DBD (arrow; transitional area)

**Fig. 6:**
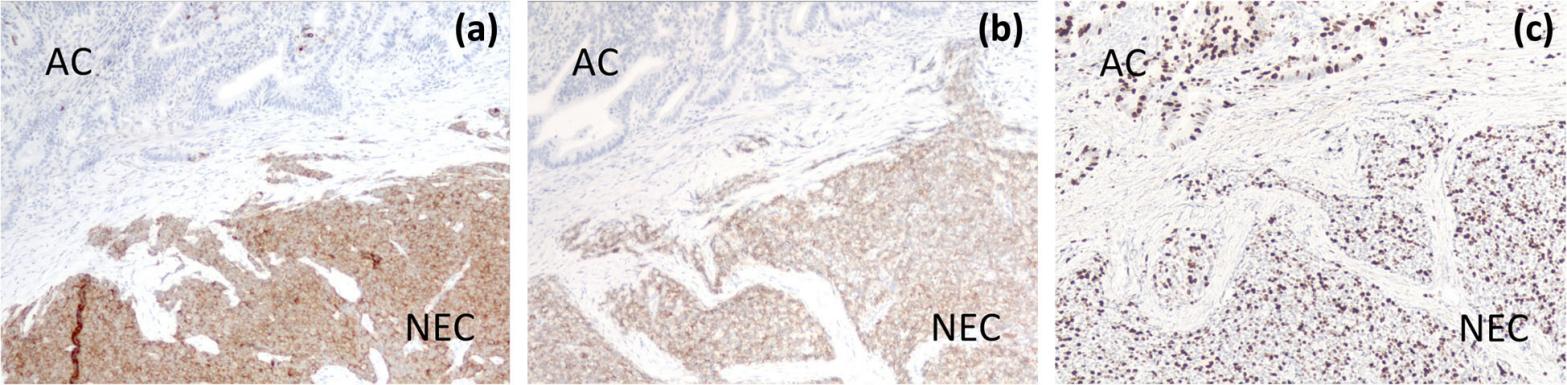
On immunohistochemical staining, NEC components were diffusely positive for **a** synaptophysin and **b** CD56, and the MIB-1 index was 30% (**c**)

**Fig. 7:**
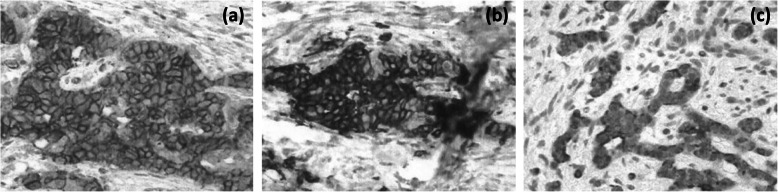
NEC component was strongly positive for **a** SSTR2, **b** SSTR5, and **c** mTOR

Under a diagnosis of primary cholangiocarcinoma (CCA) of the DBD, a subtotal stomach-preserving pancreaticoduodenectomy was performed. Neither peritoneal dissemination nor lymph node metastases were found during the operation. Macroscopically, an irregularly shaped nodular tumor was found in the DBD (Fig. 4). Microscopically, the lesion was seen to be composed of predominantly well-differentiated tubular AC in the superficial layer of the tumor, admixed with neuroendocrine carcinoma (NEC) in the deeper portion (Fig. 5), indicating a diagnosis of MANEC of the DBD. While the AC component shows papillary growth toward the lumen of the bile duct, the NEC component had infiltrated into the muscle layer, with vascular and neural invasion. Atypical epithelium was found extensively in the superficial epithelium and accessory glands of the CBD, but no malignancies were found in the gallbladder, cholecystic duct, papilla of Vater, pancreas, or duodenum; based on this, the final pathological diagnosis was MANEC of the CBD, Pat Bi, fm, pPanc0, pDu0, pHM0, pEM0, tubular, well-differentiated tubular AC>NEC, INF β, int, ly0, v0, pm1. No lymph node metastases were found. After immunohistochemical staining, NEC components were diffusely positive for synaptophysin and CD56, and the MIB-1 index was 30% (Fig. 6). In both components, p16 was positive and p53 was negative. Additionally, the NEC component was strongly positive for SSTR2, SSTR5, and mammalian target of rapamycin (mTOR) (Fig. 7).

The postoperative course was good, there were no serious complications, and he was discharged 33 days post-operation. Three months postsurgery, postoperative adjuvant chemotherapy with S-1 (120 mg/body/day) was started. More than 3 years postsurgery, he is alive without recurrence.

## Discussion

Neuroendocrine neoplasms (NENs) are composed a group of tumors exhibiting neuroendocrine phonotypes and are divided into three main categories according to the 2010 World Health Organization classification system: well-differentiated neuroendocrine tumors (NETs); grade 1 and 2, Ki-67 ≤ 20% and/or mitotic count ≤ 20 per 10 high-power fields, poorly differentiated NECs; and grade 3, Ki-67 > 20% and/or mitotic count > 20 per 10 high-power fields and MANECs [11]. Eighty percent of tumors arising in the extrahepatic biliary tract (EHBT) are well-differentiated AC; NENs are uncommon [12–21] and MANECs even more so. MANEC refers to a composite tumor characterized by coexisting glandular and neuroendocrine elements, with each accounting for > 30% of the lesion. Because of its histological complexity, the characteristics of MANEC remain poorly elucidated [9].

MANECs predominantly occur in the colon, appendix, and stomach where neuroendocrine cells are diffusely distributed [22, 23]. However, MANECs arising from the EHBT are extremely rare, with a total of 10 cases reported in the medical literature since the introduction of WHO category in 2010 (Table 1). Zhang et al. [9] reviewed the 38 cases of NEN in the EHBT, and MANECs constituted only 9 (23.7%) of the cases identified.

**Table 1:** Summary of reported cases of MANEC of the common bile duct

Author [ref]	Age	Sex	Size (cm)	Preop. diagnosis	Pathology (AC/NEC)	IHC	Treatment	Prognosis
Izumo W [1]	66	M	1.0×0.8	CCA	mode/large	CA, SP	PD	30m, alive
Komo T [2]	82	M	1.8	CCA	well/small	CA, SP	PD	7m, alive
Masui T [3]	82	M	2.5	CCA	well/small	CA, SP, CD	BDR	6M, died
Linder R [4]	82	M	1.9×1.2	CCA	poor/small	CA, SP, CD	PD	6m, alive
Onishi I [5]	74	F	2.0	IPNB	IPNB/small	SP	PD	NA
Wysocki J [6]	65	M	3.6	NA	clear/large	CA, SP, CD	BDR	5m, died
Lee SW [7]	75	M	2.0	CCA	mode/small	CA, SP, CD	BDR	11m, alive
Akhilesh SP [8]	76	M	1.4×0.8	ND	mode/small	SP, CD	BDR	NA
Zhang L [9]	64	F	4.5×3.0	CCA	poor/small	CA, SP, CD	PD	12m, died
Present case	60	M	3.0	CCA	well/small	SP, CD	PD	36m, alive

*ref* reference, *AC* adenocarcinoma, *NEC* neuroendocrine carcinoma, *IHC* immunohistochemistry, *CCA* cholangiocarcinoma, *IPNB* intraductal papillary neoplasm of the bile duct, *CA* chromogranin A, *SP* synaptophysin, *CD* cluster of differentiation 56, *BDR* bile duct resection, *PD* pancreaticoduodenectomy, *NA* not available

A high rate of misdiagnosis occurs with biliary NEN because its imaging results can appear similar to those of CCA. A well-vascularized, hypodense, and heterogeneously enhanced lesion is observed in CT scans. The common characteristics are lymph node enlargement and upstream bile duct dilation. In magnetic resonance images, biliary NENs mostly appear as nodular (45%) and intraductally growing (45%) shapes and less frequently as periductal infiltration (9%) [24]. In positron emission tomography, high glucose metabolism is usually found in NEN, especially in poorly differentiated NEC [25]. Because of the paucity of tissue obtained from ERCP brush cytology, MANEC is seldom diagnosed preoperatively. The AC component of MANEC is generally detected at the tumor surface, while the neuroendocrine component is found in the deep stroma, infiltrating the stromal and vascular tissues and lymph nodes [21]. Therefore, ERCP may fail to reach the neuroendocrine component, which is embedded in a deeper portion of the tumor [7].

Making an accurate preoperative diagnosis of biliary NEN is extremely difficult due to its indefinite clinical and imaging characteristics; as a result, most MANECs of the bile duct are initially thought to be ACs or NETs [6, 7, 26]. A previous study examined 274 cases of surgically resected biliary tract cancer specimens and reported that 13 of 53 extrahepatic bile duct cancer cases contained neuroendocrine cells and 2 were newly diagnosed as MANEC [21]. Our patient was also first diagnosed with CCA by brush cytology, probably because the AC component was localized in the superficial layer and the NEC component was located in the deeper portion.

Therefore, to make a correct pathologic diagnosis of MANEC, a surgically resected specimen with immunohistochemical staining for neuroendocrine markers may be essential for the correct diagnosis [6–9]. Of the commonly used neuroendocrine markers, two of the most reliable are synaptophysin and chromogranin. Synaptophysin, with its small clear vesicles in tumor cells, and chromogranin, with its large neurosecretory granules, are usually stained diffusely in NEN [9]. CD56 (NCAM) is also used as a neuroendocrine marker [21]. In our case, NEC components were diffusely positive for synaptophysin and CD56 on immunohistochemical staining.

The prognosis of biliary MANEC is generally poor. The natural history of these tumors is still under debate with some reporting the NEC component showing more aggressive behavior, whereas others have concluded that, if the NEC component is well-differentiated, prognosis depends on the AC component [8]. However, the NEC component is said to have a greater effect on prognosis. Zhang et al. [9] reported significant variation by pathological type in the survival outcome of patients with NEN in the EHBT. The median overall survival for patients diagnosed with NET, NEC, and MANEC was 100, 7.7, and 16.6 months, respectively. Additionally, old age and tumor recurrence were found to negatively affect clinical outcomes. The Ki-67 staining index and mitotic count are crucial for tumor grading, as defined in the classification systems [11]. Harada et al. [21] reported that NEC components showed higher proliferative activity on Ki-67 immunostaining, compared to AC components, suggesting that neuroendocrine components, particularly NEC, in biliary MANEC could determine prognosis. In this case, the Ki-67 index of the NEC component was 30%, which was higher than that of the AC component.

The treatment algorithm for MANEC is not well established [7–9]. Surgery may be a mainstay for the treatment of MANEC, and adjunctive therapy with chemotherapy, radiotherapy, and somatostatin analogs can be considered according to the NEC type [7–9]. The chemotherapy regimen selection for MANEC remains a major clinical dilemma, since it is complicated by a mixture of distinctive malignant histologies. It is reasonable to treat MANEC in accordance with the more aggressive component of the tumor. MANECs containing a well-differentiated NET component and AC component should be treated as ACs. MANECs containing a poorly differentiated NEC component should be treated as NECs. A NEN shown to possess the receptor for somatostatin (SSTR) is a good candidate for treatment with a somatostatin analog. In this case, SSTR2 and mTOR were positive in the NEC component upon immunohistochemical staining, indicating the use of somatostatin analogs as adjuvant therapy. Therefore, after tumor resection, the pathological differentiation and diagnosis of NENs is important for chemotherapy [21]. Adjuvant therapies have been seldom attempted in patients with biliary MANEC because clear, consensus-based evidence is lacking. However, adjuvant chemotherapy may be justified, as recurrent events were noted in 2/9 patients (22.2%) [9]. In this case, S-1 was administered 3 months postsurgery as adjuvant chemotherapy because the AC component was dominant in the tumor. Further studies are required to tailor chemotherapy strategies and to determine which component to target to obtain the best therapeutic benefits.

## Conclusions

Herein, we presented the case of a 60-year-old male with MANEC of the DBD. Most MANEC cases, including this one, are initially diagnosed as CCA. Because MANEC may show more aggressive behavior and have a poor prognosis, it may be important to identify the NEC component using immunohistochemical staining with neuroendocrine markers for correct diagnosis and choice of treatment.

## Data Availability

All datasets supporting the conclusions of this article are included in this published article.

## Abbreviations

MANEC: Mixed adenoneuroendocrine carcinoma
CBD: Common bile duct
DBD: Distal bile duct
CCA: Cholangiocarcinoma
AC: Adenocarcinoma
CT: Computed tomography
ERCP: Endoscopic retrograde cholangiopancreatography
NEC: Neuroendocrine carcinoma
NET: Neuroendocrine tumor
mTOR: Mammalian target of rapamycin
EHBT: Extrahepatic biliary tract
